# Semantic and spatial congruency mould audiovisual integration depending on perceptual awareness

**DOI:** 10.1038/s41598-021-90183-w

**Published:** 2021-05-25

**Authors:** Patrycja Delong, Uta Noppeney

**Affiliations:** 1 Centre for Computational Neuroscience and Cognitive Robotics, University of Birmingham, Birmingham, UK; 2 Donders Institute for Brain, Cognition and Behaviour, Radboud University, Nijmegen, The Netherlands

**Keywords:** Cognitive neuroscience, Sensory processing

## Abstract

Information integration is considered a hallmark of human consciousness. Recent research has challenged this tenet by showing multisensory interactions in the absence of awareness. This psychophysics study assessed the impact of spatial and semantic correspondences on audiovisual binding in the presence and absence of visual awareness by combining forward–backward masking with spatial ventriloquism. Observers were presented with object pictures and synchronous sounds that were spatially and/or semantically congruent or incongruent. On each trial observers located the sound, identified the picture and rated the picture’s visibility. We observed a robust ventriloquist effect for subjectively visible and invisible pictures indicating that pictures that evade our perceptual awareness influence where we perceive sounds. Critically, semantic congruency enhanced these visual biases on perceived sound location only when the picture entered observers’ awareness. Our results demonstrate that crossmodal influences operating from vision to audition and vice versa are interactively controlled by spatial and semantic congruency in the presence of awareness. However, when visual processing is disrupted by masking procedures audiovisual interactions no longer depend on semantic correspondences.

## Introduction

To create a unified percept of the multisensory environment the brain integrates information across the senses. A critical question is to what extent these multisensory interactions can arise in the absence of awareness. Leading theories of consciousness such as the Global Workspace Theory posit that only conscious information ignites a so-called global workspace that allows broadcasting of information across distant brain regions via long-range connectivity^1,2^, whereas non-conscious processing evolves mainly in local neural circuitries. Yet, the relationship between information integration and perceptual awareness remains contentious.

An extensive body of research has shown that supraliminal stimuli from one sensory modality influence processing of subliminal stimuli in another modality^3–21^. Most notably, synchronously presented sounds have been shown to ‘boost’ invisible pictures or flashes into observers’ awareness based on semantic and spatial congruency as indicated by an increase in categorization accuracy and visibility ratings^3,4,10,12,18^. These influences from supraliminal to subliminal processing are consistent with the Global Workspace Theory, because conscious signals can interact with regions in other sensory systems via long-range connectivity throughout the global workspace.

More recent evidence also indicates influences of unconscious visual signals on conscious sound perception^22^ or even cross-modal associative learning in the absence of awareness^23,24^. Notably, Delong et al. (2018) have recently presented observers with flashes and sounds in synchrony, but at variable spatial disparities under continuous flash suppression. They showed that an invisible flash can bias observers’ perceived sound location, such that it is attracted towards the invisible flash^22^, a phenomenon coined spatial ventriloquist illusion. Recent computational modelling efforts have shown that the spatial ventriloquist illusion arises from reliability-weighted integration of audiovisual spatial signals^25–27^. These results therefore suggest that invisible flashes can influence sound perception via mechanisms of audiovisual integration. By contrast, the McGurk illusion, which arises for instance by integrating a ‘ba’ phoneme with a ‘ga’ viseme into an illusionary ‘da’ percept^28^, is abolished when the facial articulatory movements are suppressed from observers’ awareness^29,30^. These discrepancies may be explained by the fact that the spatial ventriloquist illusion relies on low level spatial cues, while the McGurk illusion requires the extraction and integration of complex phonological features (i.e. visemes and phonemes).

Crucially, in everyday life several inter-sensory correspondence cues such as temporal synchrony, spatial colocation or semantic congruency^31,32^ can inform the brain about whether signals come from a common source and should be integrated into a unified percept. Previous evidence suggests that the brain does not always use all available cues to arbitrate between integration and segregation^33^. For instance, the spatial ventriloquist illusion has proven relatively immune to violations of semantic or phonological correspondences^34–37^. Only recent research that increased the complexity of the binding problem by presenting one central sound together with two bilateral faces has revealed a small effect of phonological congruency on spatial ventriloquism^38^. These weak phonological/semantic influences on spatial ventriloquism suggest that the brain prioritizes the computations of correspondence cues that are most relevant for observers’ current perceptual goals and tasks^39^. Spatial correspondences that are computed along the dorsal stream are more relevant for spatial tasks, while semantic cues that are computed along the ventral stream shape audiovisual binding for object categorization or identifications tasks^40,41^.

To summarize, previous research has shown that invisible flashes can influence where observers perceive sounds. Observers’ perceived sound location was attracted towards a concurrent, yet spatially disparate, flash, even when this flash was invisible. Put simply, the ventriloquist illusion is preserved for invisible flashes. Crucially, recent tentative evidence has shown that the spatial ventriloquist effect is enhanced when audiovisual signals are semantically congruent, suggesting that semantic correspondences enhance audiovisual binding for spatially disparate sounds.

Collectively, these results raise the important question to what extent spatial and semantic cues jointly influence audiovisual binding depending on observers’ perceptual awareness and goals. Will semantic congruency enhance the binding of spatially disparate audiovisual signals and thereby induce a stronger ventriloquist illusion even when the visual signal is rendered invisible and hence obliterated from observers’ subjective awareness?

The current study addresses these questions in two psychophysics experiments. In experiment 1 we first developed a novel paradigm that convincingly shows the impact of semantic congruency on audiovisual binding. Observers were presented with pictures and synchronous sounds that were spatially and/or semantically congruent or incongruent. On each trial they reported the location of the sound. We hypothesized that observers experience a spatial ventriloquist illusion more frequently, if auditory and visual signals were spatially disparate but semantically congruent. To render the paradigm more sensitive to subtle effects of semantic congruency we increased the complexity of the binding problem by presenting either one or two pictures (i.e. unilateral vs. contralateral presentation mode, see discussion above and^38^).

Experiment 2 manipulated the spatial and semantic congruency of the pictures and sounds using the same design as experiment 1. Importantly, it employed forward–backward masking to obliterate visual awareness on a fraction of trials allowing us to compare spatial ventriloquism for physically identical pictures that differed in their visibility. On each trial observers located the sound, identified the picture and rated its visibility using the Perceptual Awareness Scale^42^. First, we investigated whether semantic and spatial correspondences jointly ‘boost’ a picture into observers’ awareness as indicated by increases in their visibility scores and picture identification accuracy. Second, we assessed whether spatial and semantic congruency influence observers’ perceived sound location depending on the visibility of the picture.

In this study we focused selectively on observers’ subjective rather than objective awareness. In other words, we refer to visual stimuli as invisible, unconscious or unaware, if observers subjectively rated them as ‘invisible’, irrespective of whether these invisible visual pictures are associated with chance performance on the picture identification task. This is an explicit experimental choice that needs to be justified, because both objective and subjective awareness criteria have their strengths and limitations (for further discussion see^43–47^). Subjective thresholds reflect observers’ phenomenal experience, but are susceptible to criterion shifts driven by observers’ confidence^48^. For instance, observers may judge stimuli as ‘invisible’ because they set a high criterion and judge stimuli as visible only when they are perceived with a high level of confidence. As a result, ‘subjectively invisible’ stimuli may enable better than chance performance accuracy on perceptual or other tasks.

Conversely, objective criteria of awareness define stimuli as unconscious or unaware when they are associated with chance performance. Objective awareness criteria have been criticised for being too strict, focusing on unconscious processing of degraded stimuli^49^. Moreover, numerous studies that employed objective awareness criteria relied on post-hoc selection of trials, conditions or participants based on ‘chance performance’. They are thus confounded by serious statistical biases resulting from regression towards the mean (see^50^ for comprehensive list of past research). The basic strategy of those studies is to apply two tasks concurrently and post-hoc select conditions, trials or participants based on chance performance on task 1 while demonstrating better than chance performance on task 2. Based on this dissociation, it is then argued that the cognitive processes in task 2 can be performed ‘unconsciously’, because chance performance was obtained on task 1. The fallacy of this inferential strategy is that performance accuracy measured on task 1 is only a noisy and uncertain estimate of observers’ true performance. In other words, the performance measure depends on observers’ true performance and additional measurement error. Hence, conditions, items or participants that have been post-hoc selected because of their chance performance (i.e. extremely low performance) are likely to show higher and even better than chance performance when tested again (i.e. regression towards the mean). In short, post-hoc selection of participants, conditions, sessions, items, trials etc. based on chance performance does not guarantee true chance performance. To avoid problems associated with regression towards the mean, studies would need to select conditions, items or participants based on a separate data set for instance using strategies of crossvalidation.

Obviously, regression towards the mean is a generic statistical phenomenon. Thus, selecting participants post-hoc based on a subjective (un)awareness criterion (e.g. subjects with 100% of trials judged invisible) is also conflated by regression towards the mean. However, subjective awareness criteria are rarely used to infer that a subset of participants is able to perform a task in the absence of awareness. Instead, research focusing on subjective awareness criteria treats the trial-specific dependent variable ‘visibility’ as an independent factor, acknowledging that observers’ visibility judgments may vary across trials because of changes in sensory processing, decisional criteria or simply noise. Indeed, this study follows exactly this line of reasoning and recognizes that differences in other dependent measures for trials judged visible or invisible may arise at multiple levels.

Finally, as previously argued^23^, objective criteria are less appropriate than subjective criteria of awareness to test global workspace theory. First, the global workspace theory postulates that stimuli entering the global workspace are available for visibility report thereby intimately linking global workspace predictions with subjective criteria of awareness. Second, processing of subliminal stimuli in local neural circuitries can enable better than chance performance even though these stimuli do not enter the global workspace. Therefore, better than chance performance, i.e. a violation of the objective awareness criterion, does not necessary imply that stimuli accessed the global workspace. These considerations suggest that subjective awareness measures (i.e. Perceptual Awareness Scale^42^) are more suitable for addressing the questions of our current study.

## Methods

### Participants

After giving informed consent, 44 healthy young adults (mean age ± std: 20.9 ± 5.7 years, range: 18–47 years, 6 male, 8 left-handed, 2 ambidextrous) took part in experiment 1, 44 subjects (mean age ± std: 20.9 ± 2.2 years, range: 18–30 years, 7 male, 4 left-handed, 2 ambidextrous) in experiment 2. 12 of those subjects took part in both experiments. The study was performed in accordance with the principles outlined in the Declaration of Helsinki and was approved by the local (STEM) ethics review board of the University of Birmingham.

The sample size (n = 44) was determined to allow the detection of moderate (Cohen’s d = 0.5, for paired two-tailed t-test) semantic influences on the ventriloquist effect with a statistical power of 0.9.

### Stimuli and apparatus

Visual stimuli were a selection of six pictures (bird, car, dog, guitar, phone and daffodil) from the Bank of Standard Stimuli database^51,52^, normalized for their familiarity. Images were displayed for 24 ms on white background (mean luminance 11 cd/m^2^). On each trial, a square image (5 visual degree width) was presented at ± 2.5 visual angle along the azimuth from the centre of the screen.

Auditory stimuli were five sounds (bird, car, dog, guitar, phone) downloaded from http://www.findsounds.com (on 26/07/2017). The sounds were edited to a fixed duration of 150 ms. Peak amplitudes of all sounds were equalized with Audacity software (http://audacityteam.org). The sounds were presented via circumaural headphones (Sennheiser HD 280 Pro, presented at 66–75 dB SPL). To create a virtual auditory spatial signal with binaural (interaural time and amplitude differences) and monoaural spatial filtering cues, the sounds were convolved with spatially specific head-related transfer functions (HRTFs, MIT Media Lab database^53^) interpolated to the specific spatial locations.

Psychophysical stimuli were generated and presented on a PC with Windows XP and Psychtoolbox version 3.0.11^54,55^ running on MATLAB R2014a (Mathworks Inc., Natick, Massachusetts).

Participants sat in dimly lit room in front of a computer screen at viewing distance of 90 cm. Visual stimuli were presented on a CRT monitor at a frame rate of 85 Hz (NVIDIA Quadro FX 380 graphics card). Auditory stimuli were digitized at a sampling rate of 44.8 kHz and presented with Sound Blaster Z SB1500 sound card.

### Experimental design and procedure

Both psychophysics experiments employed a spatial ventriloquist paradigm. Participants were presented with a sound at 2.5° or − 2.5° along the azimuth together with one or two pictures at 2.5° and/or − 2.5° (i.e. unilateral vs. bilateral presentation mode). In bilateral presentation mode one picture (i.e. distractor picture) was always a daffodil which does not produce any sounds in our natural environment, while the other picture (i.e. target picture) was selected from the remaining set of five pictures that are naturally associated with a specific source sound. We manipulated whether the single picture (in the unilateral mode) or the target picture (in the bilateral mode) was i. spatially collocated (i.e. same hemifield) or disparate (i.e. opposite hemifield) and ii. semantically congruent or incongruent with the sound. In the semantically congruent condition, the picture was presented together with the corresponding sound (5 congruent stimuli pairs). In the semantically incongruent condition, the picture was presented with one of the four other sounds (20 combinations of incongruent pairs). Each picture and sound were presented equally often in each of the conditions. In short, experiments 1 and 2 conformed both to a factorial design manipulating 2 (AV spatial collocation: collocated, disparate) × 2 (AV semantic congruency: congruent, incongruent) × 2 (visual presentation mode: unilateral, bilateral pictures). The key difference between experiment 1 and 2 was that experiment 2 used forward–backward masking to manipulate the visibility of the picture.

#### Experiment 1

**Figure 1:**
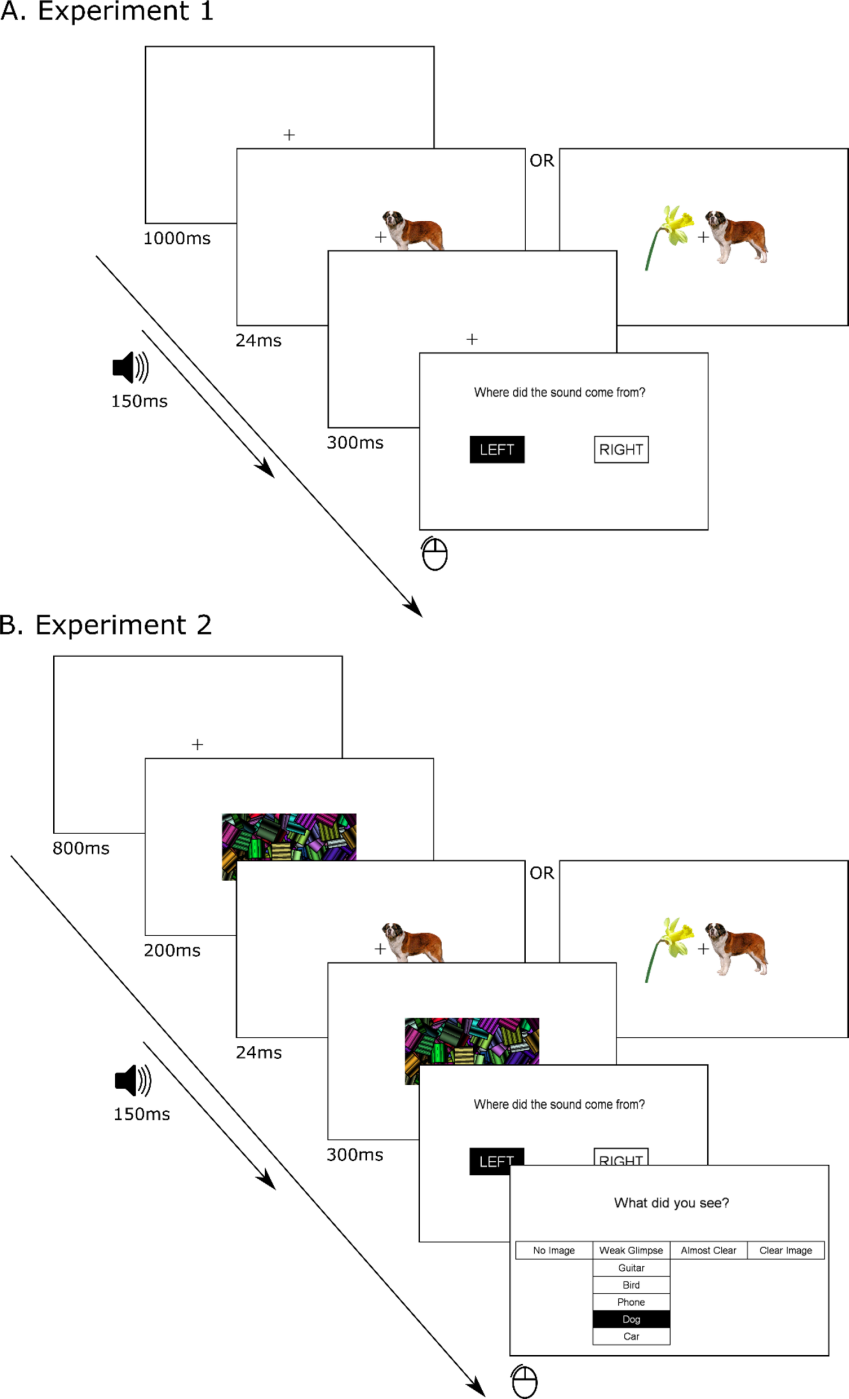
Example trials for unilateral and bilateral presentations. In unilateral visual presentations the picture of daffodil was not shown. (**A**) Experiment 1—without visual masking. (**B**) Experiment 2—with forward–backward masking.

Each trial started with the presentation of a fixation cross for 1000 ms. Next, the target picture was displayed for a duration of 24 ms, followed by the presentation of a white screen with a fixation cross for 300 ms. The sound was presented in synchrony with the picture. In the bilateral presentation mode the distractor image (daffodil) was displayed in the hemifield opposite to the target picture (see Fig. 1A). After each presentation participants were asked to locate the sound (left vs right), by shifting the cursor to the relevant response box that was displayed on the screen (the selected answer was highlighted) and pressing the left mouse button. The response screen was presented until the answer was provided or up to a maximum of 5 s.

Experiment 1 consisted of two blocks: 320 trials with unilateral and 320 trials with bilateral image presentation. The order of the blocks was counterbalanced across subjects. The remaining factors/conditions were randomized across trials. In total the experiment included 80 trials per condition × 2 (spatially collocated vs. disparate) × 2 (semantically congruent vs. incongruent) × 2 (unilateral vs. bilateral picture presentation) = 640 trials.

#### Experiment 2

Experiment 2 had the same experimental design as experiment 1, but employed forward–backward masking to suppress participants’ awareness of the visual stimuli. The mask was a Mondrian consisting of rectangles filled with coloured, dynamically moving square gratings (similar as in^22,56^), which changed their colour and position randomly at a frequency of 10.6 Hz. Each grating’s texture was shifted every 11.8 ms (i.e. each frame of the monitor with 85 Hz refresh rate) to generate apparent motion.

Each trial started with the presentation of a fixation cross for 800 ms, followed by the presentation of the mask for 200 ms. Next the target image was displayed on the screen for 24 ms together with the synchronous sound. Immediately after the visual stimulus a second mask was presented for 300 ms.

After each presentation participants reported 1. the sound location and 2. the semantic category of the picture together with 3. a picture visibility rating (using 4 level Perceptual Awareness Scale^42^). Critically, participants reported visibility and semantic category at the same time (see Fig. 1B) using the mouse cursor (the selected box was highlighted). They confirmed their selection by pressing the left mouse button. The response screen was presented until the answer was provided or up to a maximum of 5 s (measured from the onset of sound localization screen).

Experiment 2 consisted of two blocks: 640 trials with unilateral and 640 trials with bilateral image presentation. The order of the blocks was counterbalanced across subjects. The remaining factors/conditions were randomized across trials. In total the experiment included 160 trials per condition × 2 (spatially collocated vs. disparate) × 2 (semantically congruent vs. incongruent) × 2 (unilateral vs. bilateral picture presentation) = 1280 trials.

Prior to the experiment subjects performed a practice session consisting of 5 unmasked visual trials, on which they were asked to identify the picture. If accuracy was lower than 100%, the practice session was repeated. This was to familiarise subjects with the combined task of reporting semantic category and rating picture visibility.

### Data analysis

The descriptive statistics for sound localization accuracy, picture identification accuracy and visibility ratings for each participant and condition were computed in MATLAB R2016b (Mathworks, Natick, Massachusetts). We report the number of trials for each condition (across subjects’ mean ± SD) in supplementary tables S5 and S6.

Generalized linear (GLMM) and cumulative link mixed (CLMM) models were fitted using lme4^57^ and ordinal^58^ packages in R version 3.6.3^59^. For each GLMM/CLMM we first defined the random effects for each model. We assessed models including from none to up to three random effects: subject identity, picture type (e.g. dog, bird) and sound type. Model selection was based on the Akaike Information Criterion. Random effects selected for each model are specified in the sections below. R code for the fitted models is available in the OSF repository. Simple main effects for hypothesis driven interactions were computed using emmeans package in R. Following reviewers’ and the editor’s suggestions, we report original p-values and p-values adjusted for all possible main effects and interactions in each GLMM. Additional exploratory analyses of all contrasts are reported with Benjamini–Hochberg correction for multiple comparisons in the supplementary material S1.

Bayes Factors were computed using brms package in R^60^. Bayesian models (using the same model formulas as described GLMMs) were fitted which did or did not include the effect of interest and Bayes Factors comparing these models were computed using *bayes_factor* function. Please note BF_10_ quantifies the evidence for the alternative relative to the null-hypothesis. Conversely, BF_01_ quantifies the evidence for the null- relative to the alternative hypothesis. Bayes factors between 1 and 3 (or 1/3 and, 1 respectively) are considered indecisive. Bayes factors above 3 provide substantial evidence.

#### Experiment 1

We entered sound localization accuracy for each trial (i.e. coded as 1 or 0) and participant into generalized linear mixed effects model (binomial distribution, logit link function). The model included spatial collocation (collocated = 1, disparate = 0), semantic congruency (congruent = 1, incongruent = 0) and visual presentation mode (unilateral = 0, bilateral = 1) and their two-way and three-way interactions as fixed effects and subject identity and sound type as random effects.

#### Experiment 2

Perceptual Awareness Scale ratings were transformed into ordinal numbers from 1 to 4 (No Image = 1, Weak Glimpse = 2, Almost Clear = 3, Clear Image = 4). We entered visibility ratings for each trial and participant into a cumulative link mixed model (CLMM) for ordinal data. The model included spatial collocation (collocated = 1, disparate = 0), semantic congruency (congruent = 1, incongruent = 0) and visual presentation mode (unilateral = 0, bilateral = 1) and their two and three way interactions as fixed effects and subject identity, picture type and sound type as random effects.

We entered picture identification accuracy and sound localization accuracy for each trial and participant into generalized linear mixed effects models (binomial distribution, logit link function). To assess the extent to which audiovisual binding and the impact of semantic congruency depend on the visibility of the picture, we treated visibility as an independent factor. For this, we classified trials with “No Image” ratings as ‘invisible’ and the other three visibility ratings (Weak Glimpse, Almost Clear, Clear Image) as ‘visible’. Hence, the models included spatial collocation (collocated = 1, disparate = 0), semantic congruency (congruent = 1, incongruent = 0), visual presentation mode (unilateral = 0, bilateral = 1) and visibility (visible = 1, invisible = 0) and their two-way and higher order interactions as fixed effects and subject identity and sound type as random effects in both models. For picture identification accuracy, the model also included picture type as random effect.

## Results

Across all result sections, the text reports only those results that pertain to our specific hypotheses. For complete results, we refer the reader to the tables, which provide the results pertaining to our hypotheses in roman and the remaining results in italics. The tables report both the original p-values and p-values that have been adjusted for the total number of main effects and interactions within each generalized linear mixed effects model.

### Experiment 1

#### Hypotheses

We expected a greater sound localization accuracy for spatially congruent relative to incongruent signals. Moreover, the effect of spatial congruency should be enhanced for semantically congruent relative to incongruent signals (i.e. interaction) indicating that semantic congruency enhances audiovisual binding. Further, the spatial congruency effect and this interaction should be stronger for bilateral presentation mode^38^.

#### Results

**Figure 2:**
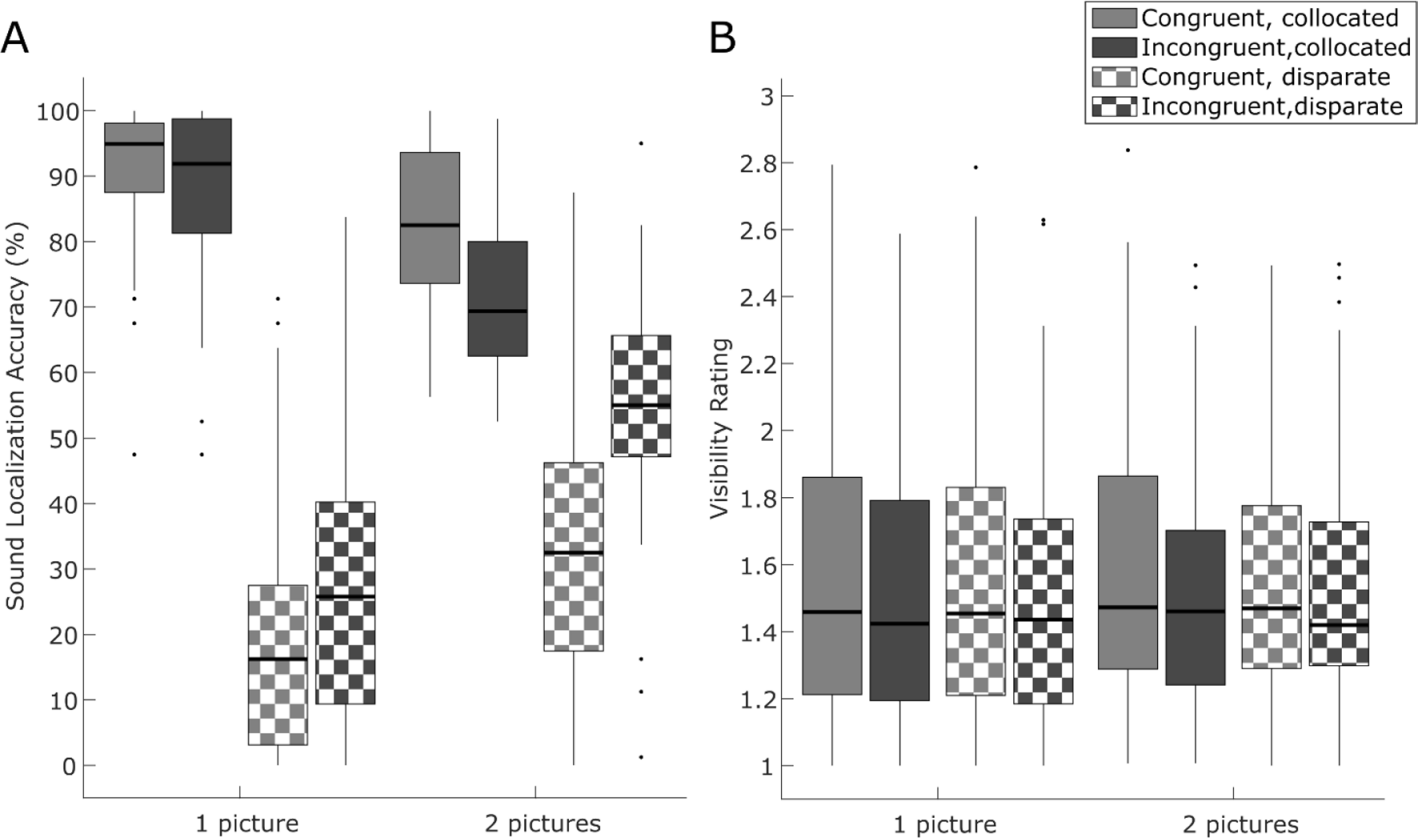
Sound localization (Experiment 1) and visibility ratings (Experiment 2). Box plots show interquartile range, with whiskers extending to most extreme data points excluding outliers, which are plotted individually. Thick lines in each plot indicate median values. (**A**) Experiment 1 (without visual masking): sound localization accuracy (left vs right). (**B**) Experiment 2 (with forward–backward masking): perceptual awareness scale rating transformed to numerical values: 1—not seen, 2—weak glimpse, 3—almost clear, 4—clear image.

Consistent with our hypotheses, we observed a significant effect of spatial congruency. The odds of correct sound localization were 22.3 times higher for collocated than disparate audiovisual stimuli (coefficient = 3.1 ± 0.07, p < 0.001), indicating that visual signals influence observers’ spatial sound perception (see summary of GLMM in Table 1, box plots of subject accuracies in Fig. 2A). The effect of spatial congruency most likely results from two mechanisms. First, integration of collocated audiovisual signals increases the precision of spatial representations and thereby sound localization accuracy^27,61^. Second, a spatially disparate visual signal biases observers’ perceived sound location, i.e. induces a spatial ventriloquist illusion, which decreases sound localization accuracy^25^.

**Table 1:** Summary of the GLMM fitted for Sound localization accuracy in Experiment 1 (unmasked pictures).

Predictor	Coefficient	Std. error	95% CI		z value	p value	adj. p
(Intercept)	*− 1.02*	*0.1*	*− 1.22*	*− 0.83*	*− 10.238*	*** < 0.001***	*** < 0.001***
Spatial congruency	3.1	0.07	2.98	3.23	47.147	** < 0.001**	** < 0.001**
Semantic congruency	*− 0.42*	*0.06*	*− 0.54*	*− 0.31*	*− 7.259*	*** < 0.001***	*** < 0.001***
Presentation mode	*1.25*	*0.05*	*1.14*	*1.35*	*23.759*	*** < 0.001***	*** < 0.001***
Spatial congruency * Semantic congruency	0.65	0.1	0.46	0.84	6.678	** < 0.001**	** < 0.001**
Spatial congruency * Presentation mode	− 2.35	0.08	− 2.52	− 2.19	− 28.206	** < 0.001**	** < 0.001**
Semantic congruency * Presentation mode	*− 0.47*	*0.08*	*− 0.62*	*− 0.32*	*− 6.104*	*** < 0.001***	*** < 0.001***
Spatial congruency * Semantic congruency * Presentation mode	0.93	0.12	0.68	1.17	7.438	** < 0.001**	** < 0.001**

The results pertaining to our hypotheses are shown in roman, the remaining results in italics. Bold indicates significant p-values.

Importantly, we also observed a significant interaction between spatial and semantic congruency (coefficient = 0.65 ± 0.1, p < 0.001). Semantic congruency increased participants’ sound localization accuracy for spatially congruent trials (coefficient = 0.45 ± 0.05, p < 0.001), but decreased their accuracy for spatially incongruent trials (coefficient = − 0.66 ± 0.04, p < 0.001), reflecting a stronger ventriloquist effect (see Table 1, see Fig. 2A for box plots of subjects’ accuracies and supplementary materials S1 for means and standard errors for each condition). In short, semantic congruency enhanced audiovisual binding and thereby amplified the influence of the picture’s location on observers’ perceived sound location.

It may be surprising that even in the bilateral presentation mode a semantically incongruent target picture ‘boosts’ observers’ sound localization accuracy when it is collocated relative to non-collocated with the sound. Why does the brain treat a semantically incongruent target picture different from a neutral ‘daffodil’? We suspect that the target picture attracts observers’ perceived sound location more than the concurrent distractor picture because it varies across trials and is thus more salient^62^. Indeed, previous research on saliency maps has shown that novel events are more salient^63^. Potentially, target pictures may also be more salient because they may automatically activate representations in the auditory and visual systems. While our study was not designed to disentangle these different explanations, it is important to emphasize that this is irrelevant for assessing our questions of interest. Because the same distractor picture was presented throughout the entire experiment, our effects of interest are not confounded. In fact, the bilateral presentation mode can be considered a within study replication of the unilateral presentation mode. It was introduced, because previous research suggested that semantic congruency may be more effective in increasing the ventriloquist effect under bilateral presentation mode.

Indeed, as expected we observed a significant interaction (coefficient: 0.93 ± 0.12, p < 0.001) between spatial and semantic congruency with presentation mode (bilateral = 1, unilateral = 0). The effect of semantic congruency on audiovisual binding is stronger for bilateral presentation mode (congruency effect in bilateral presentation mode: 1. collocated stimuli: coefficient = 0.68 ± 0.06, p < 0.001, 2. disparate stimuli: coefficient = − 0.9 ± 0.05, p < 0.001; in unilateral presentation mode: 1. collocated stimuli: coefficient = 0.23 ± 0.08, p = 0.004, 2. disparate stimuli: coefficient = − 0.42 ± 0.06, p < 0.001), which indicates that the impact of semantic congruency on audiovisual binding is more prominent when the complexity of the binding problem and thereby observers’ uncertainty about the underlying causal structure is increased.

### Experiment 2

#### Visibility rating

##### Hypotheses

We expected that audiovisual binding boosts flashes into observers’ awareness as reflected in greater visibility for spatially congruent relative to incongruent and for semantically congruent relative to incongruent signals^3,4,12^. Moreover, we expected a significant interaction: the effect of spatial congruency on visibility should be enhanced when audiovisual signals are semantically congruent.

##### Results

**Figure 3:**
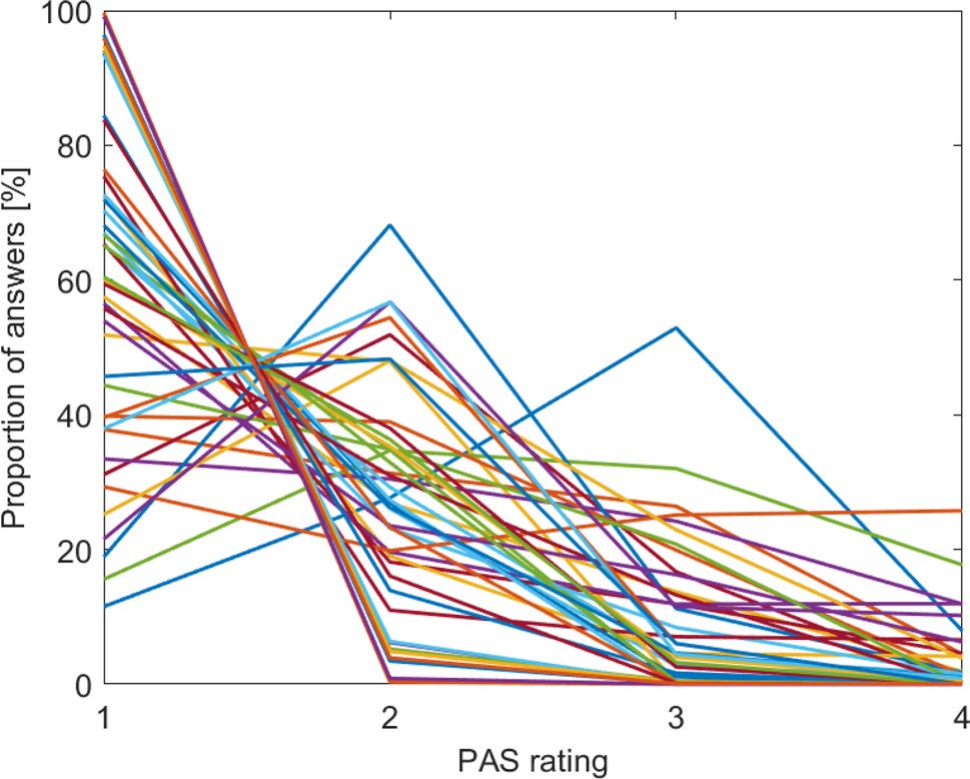
Visibility rating (Experiment2). Figure shows proportions of perceptual awareness scale (PAS) ratings: 1—not seen, 2—weak glimpse, 3—almost clear, 4—clear image. Each line represents an individual participant.

We observed a non-significant trend for greater visibility rating for semantically congruent relative to incongruent pictures (coefficient = 0.07 ± 0.04, p = 0.054, with indecisive BF_10_ = 0.52; see summary of CLMM in Table 2, box plots of subjects’ visibility ratings in Fig. 2B, supplementary materials S1 for means and standard errors for each condition and individual distributions of PAS ratings for individual subjects in Fig. 3). Moreover, we observed a non-significant trend for an interaction between spatial and semantic congruency (coefficient = 0.09 ± 0.05, p = 0.097, with indecisive BF_10_ = 0.57). Even though these effects are only non-significant trends and when we further adjust them for the total number of all possible main effects and interactions in the generalized linear mixed effects model they are even less significant (see Table 2, last column for adjusted p-values), we report them for completeness, as they replicate earlier findings of congruency effects on visibility ratings^3,4,11–13,19^. However, contrary to our hypothesis, we did not observe an effect of spatial congruency (coefficient = -0.04 ± 0.04, p = 0.3), with Bayes Factor providing substantial evidence for the null relative to the alternative hypothesis (BF_01_ = 7.03).

**Table 2:** Summary of the CLMM fitted for visibility rating in Experiment 2 (masked pictures).

Predictor	Coefficient	Std. error	95% CI		z value	p value	adj. p
(Intercept—rating 1|2)	*0.81*	*0.42*	*− 0.01*	*1.62*	*1.935*	*0.053*	*0.135*
(Intercept—rating 2|3)	*2.99*	*0.42*	*2.17*	*3.8*	*7.173*	*** < 0.001***	*** < 0.001***
(Intercept—rating 3|4)	*4.77*	*0.42*	*3.95*	*5.59*	*11.437*	*** < 0.001***	*** < 0.001***
Spatial congruency	− 0.04	0.04	− 0.11	0.04	− 1.036	0.3	0.498
Semantic congruency	0.07	0.04	0	0.15	1.928	0.054	0.135
Presentation mode	*0.03*	*0.04*	*− 0.05*	*0.1*	*0.722*	*0.471*	*0.588*
Spatial congruency * Semantic congruency	0.09	0.05	− 0.02	0.2	1.661	0.097	0.193
Spatial congruency * Presentation mode	*0.05*	*0.05*	*− 0.06*	*0.16*	*0.937*	*0.349*	*0.498*
Semantic congruency * Presentation mode	*0.01*	*0.05*	*− 0.09*	*0.12*	*0.201*	*0.841*	*0.844*
Spatial congruency * Semantic congruency * Presentation mode	*− 0.01*	*0.08*	*− 0.16*	*0.13*	*− 0.197*	*0.8444*	*0.844*

The results pertaining to our hypotheses are shown in roman, the remaining results in italics. Bold indicates significant p-values.

#### Picture identification accuracy

##### Hypotheses

We expected picture identification accuracy to be greater on visible than invisible trials. Further, spatial and semantic congruency should increase picture identification accuracy – possibly in an interactive fashion. Further, the effect of semantic congruency and possibly the interaction should be stronger for invisible than visible trials (i.e. interaction between visibility and semantic congruency).

##### Results

**Figure 4:**
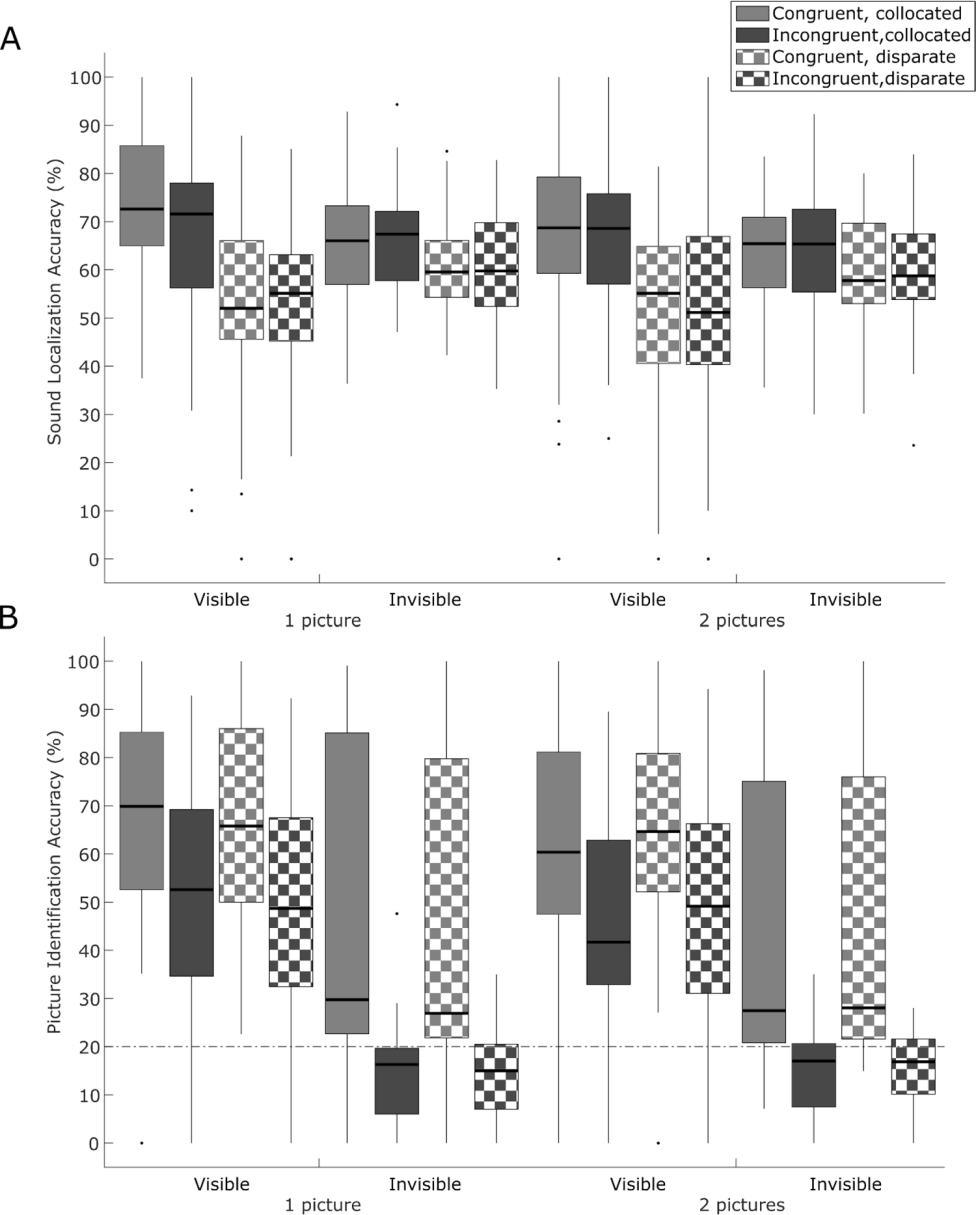
Sound localization and picture identification accuracies (Experiment 2). Box plots show interquartile range, with whiskers extending to most extreme data points excluding outliers, which are plotted individually. Thick lines in each plot indicate median values. (**A**) Sound localization accuracy (Left vs Right). (**B**) Picture identification accuracy (choice of 1 out of 5). Dashed line denotes chance level of 20%.

As expected, participants were 6.8 times more likely to identify visible compared with invisible pictures (effect of visibility, coefficient = 1.92 ± 0.06, p < 0.001). Further, we observed a significant main effect of semantic congruency. Observers were 8 times more likely to identify pictures correctly when they were presented together with semantically congruent sounds (coefficient = 2.07 ± 0.06, p < 0.001). Critically, we also observed a significant negative interaction between semantic congruency and visibility (coefficient = − 1.52 ± 0.08, p < 0.001). As expected observers benefitted more from semantically congruent (relative to incongruent) sounds, when the picture was invisible as indicated by higher positive coefficient of simple main effect of semantic congruency (semantic congruency effect for 1. invisible trials: coefficient = 1.83 ± 0.03, p < 0.001, 2. visible trials: coefficient = 0.63 ± 0.03, p < 0.001). In fact, as shown in Fig. 4B, picture identification for invisible trials was even below chance, when audiovisual signals were semantically incongruent. This suggests that the effect of semantic congruency on picture identification accuracy is driven at least partially by participants’ response bias, i.e. their tendency to respond according to the sound category when the picture is invisible (n.b. this strategy would lead to accuracy approaching 100% for semantically congruent presentations and approaching 0% for semantically incongruent presentations; moreover, performance in semantically con/incongruent conditions would be negatively correlated). Consistent with this conjecture, identification accuracy of semantically congruent and incongruent conditions was negatively correlated across participants for invisible trials (Pearson’s R = − 0.846, p < 0.001) and positively correlated for visible trials (Pearson’s R = 0.802, p < 0.001).

Contrary to our hypotheses, we did not observe a significant main effect of spatial congruency or an interaction between spatial and semantic congruency (see summary of GLMM in Table 3, box plots of subject accuracies in Fig. 4B and supplementary materials S1 for means and standard errors for each condition).

**Table 3:** Summary of the GLMM fitted for Picture identification accuracy in Experiment 2 (masked pictures).

Predictor	Coefficient	Std. error	95% CI		z value	p value	Adj. p
(Intercept)	*− 1.98*	*0.25*	*− 2.46*	*− 1.49*	*− 8.014*	*** < 0.001***	*** < 0.001***
Spatial congruency	0.05	0.06	− 0.08	0.17	0.712	0.477	0.816
Semantic congruency	2.07	0.06	1.96	2.18	36.615	** < 0.001**	** < 0.001**
Presentation mode	*0.24*	*0.06*	*0.12*	*0.37*	*3.789*	** < 0.001**	**< 0.001**
Visibility	1.92	0.06	1.79	2.04	30.417	** < 0.001**	** < 0.001**
Spatial congruency * Semantic congruency	− 0.02	0.08	− 0.17	0.14	− 0.21	0.834	0.937
Spatial congruency * Presentation mode	*− 0.06*	*0.09*	*− 0.23*	*0.12*	*− 0.659*	*0.51*	*0.816*
Spatial congruency * Visibility	*− 0.01*	*0.09*	*− 0.18*	*0.16*	*− 0.079*	*0.937*	*0.937*
Semantic congruency * Presentation mode	*− 0.5*	*0.08*	*− 0.65*	*− 0.35*	*− 6.387*	*** < 0.001***	*** < 0.001***
Semantic congruency * Visibility	− 1.52	0.08	− 1.68	− 1.36	− 18.717	** < 0.001**	** < 0.001**
Presentation mode * Visibility	*− 0.38*	*0.09*	*− 0.55*	*− 0.22*	*− 4.479*	*** < 0.001***	*** < 0.001***
Spatial congruency * Semantic congruency * Presentation mode	*0.05*	*0.11*	*− 0.16*	*0.27*	*0.486*	*0.627*	*0.911*
Spatial congruency * Semantic congruency * Visibility	0.02	0.11	− 0.21	0.24	0.135	0.892	0.937
Spatial congruency * Presentation mode * Visibility	*0.03*	*0.12*	*− 0.21*	*0.26*	*0.215*	*0.83*	*0.937*
Semantic congruency * Presentation mode * Visibility	*0.66*	*0.11*	*0.44*	*0.88*	*5.821*	*** < 0.001***	*** < 0.001***
Spatial congruency * Semantic congruency * Presentation mode * Visibility	*− 0.05*	*0.16*	*− 0.36*	*0.26*	*− 0.318*	*0.751*	*0.937*

The results pertaining to our hypotheses are shown in roman, the remaining results in italics. Bold indicates significant p-values.

#### Sound localization

##### Hypotheses

We expected similar results as in experiment 1 for visible trials. Consistent with our previous study^22^, the effect of spatial congruency should also be preserved for invisible trials and yet amplified for visible trials (i.e. interaction between visibility and spatial congruency). By contrast, semantic congruency should influence audiovisual binding mainly for visible trials (i.e. a three way interaction between spatial congruency, semantic congruency and visibility). A key question was whether the interaction between spatial and semantic congruency is preserved for invisible trials.

##### Results

We replicated the main effect of spatial congruency (coefficient of spatial congruency = 0.21 ± 0.05, p < 0.001), indicating that task-irrelevant visual signals influence observers’ perceived sound location even under forward–backward masking (i.e. reduced, but not zero visibility). Further, as expected the effect of spatial congruency was enhanced for visible than invisible trials (i.e. significant interaction between spatial congruency and visibility: 0.45 ± 0.07, p < 0.001). Critically, a subsequent analysis including only invisible trials confirmed the effect of spatial congruency even for invisible trials (coefficient of spatial congruency = 0.22 ± 0.05, p < 0.001). Thus, consistent with our previous study^22^, invisible flashes can influence where we perceive sounds (see summary of GLMM in Table 4, box plots of subject accuracies in Fig. 4A and supplementary materials S1 for means and standard errors for each condition).

**Table 4:** Summary of the GLMM fitted for Sound localization accuracy in Experiment 2 (masked pictures).

Predictor	Coefficient	Std. error	95% CI		z value	p value	Adj. p
(Intercept)	*0.48*	*0.09*	*0.3*	*0.66*	*5.222*	**< 0.001**	** < 0.001**
Spatial congruency	0.21	0.05	0.12	0.3	4.685	** < 0.001**	** < 0.001**
Semantic congruency	*0.02*	*0.05*	*− 0.07*	*0.11*	*0.501*	*0.616*	*0.896*
Presentation mode	*− 0.07*	*0.05*	*− 0.16*	*0.02*	*− 1.524*	*0.128*	*0.340*
Visibility	*− 0.36*	*0.05*	*− 0.46*	*− 0.25*	*− 6.867*	*** < 0.001***	*** < 0.001***
Spatial congruency * Semantic congruency	*− 0.05*	*0.06*	*− 0.18*	*0.08*	*− 0.782*	*0.434*	0.896
Spatial congruency * Presentation mode	*− 0.03*	*0.06*	*− 0.16*	*0.09*	*− 0.507*	*0.612*	*0.896*
Spatial congruency * Visibility	0.45	0.07	0.31	0.6	6.11	** < 0.001**	** < 0.001**
Semantic congruency * Presentation mode	*0.02*	*0.06*	*− 0.11*	*0.14*	*0.295*	*0.768*	*0.906*
Semantic congruency * Visibility	*− 0.04*	*0.07*	*− 0.18*	*0.1*	*− 0.596*	*0.551*	*0.896*
Presentation mode * Visibility	*0.01*	*0.07*	*− 0.13*	*0.15*	*0.118*	*0.906*	*0.906*
Spatial congruency * Semantic congruency * Presentation mode	*− 0.05*	*0.09*	*− 0.23*	*0.13*	*− 0.575*	*0.565*	*0.896*
Spatial congruency * Semantic congruency * Visibility	0.24	0.1	0.03	0.45	2.286	**0.022**	**0.071**
Spatial congruency * Presentation mode * Visibility	*0.02*	*0.1*	*− 0.18*	*0.23*	*0.225*	*0.822*	*0.906*
Semantic congruency * Presentation mode * Visibility	*− 0.02*	*0.1*	*− 0.22*	*0.18*	*− 0.185*	*0.853*	*0.906*
Spatial congruency * Semantic congruency * Presentation mode * Visibility	*− 0.06*	*0.15*	*− 0.34*	*0.23*	*− 0.383*	*0.702*	*0.906*

The results pertaining to our hypotheses are shown in roman, the remaining results in italics. Bold indicates significant p-values.

By contrast, we did not observe a significant interaction between spatial and semantic congruency or between spatial and semantic congruency and visual presentation mode suggesting that the influence of semantic congruency may not persist for invisible trials. Indeed, we observed a significant three-way interaction between semantic and spatial congruency and visibility (interaction coefficient = 0.24 ± 0.1, p = 0.022). Even after adjusting for the total number of main effects and interactions, we observed a p-values of 0.07, i.e. 0.035 when taking into account our directed hypothesis and the Bayes Factor provided substantial evidence for the alternative relative to the null hypothesis (BF_10_ = 3.85). Follow-up separate analyses (fitting the same model) for only visible trials showed a significant interaction between spatial and sematic congruency (interaction coefficient = 0.19 ± 0.08, p = 0.023). Even after adjusting for the total number of main effects and interactions, we observed a p-values of 0.094, i.e. 0.047 when taking into account our directed hypothesis and the Bayes Factor provided anecdotal evidence for the alternative relative to the null hypothesis (BF_10_ = 2.63). Simple main effects of semantic congruency for visible trials showed a similar pattern as in the experiment 1: a significant increase in accuracy for collocated trials (coefficient = 0.12 ± 0.04, p = 0.006), but an insignificant decrease for spatially disparate trials (coefficient = − 0.02 ± 0.04, p = 0.669). By contrast, for invisible trials interaction between spatial and semantic congruency was not significant (coefficient = − 0.05 ± 0.06, p = 0.427) and Bayes Factor provided substantial evidence for the null- relative to the alternative hypothesis (BF_01_ = 4.99) for its absence.

Collectively, our results demonstrate that pictures that evade observers’ subjective visual awareness can influence where we perceive sounds—yet, this audiovisual spatial binding is not modulated by semantic congruency unless the picture is visible.

## Discussion

In everyday life the brain is bombarded with many different signals. Several cross-sensory correspondence cues such as temporal synchrony, co-location or semantic correspondences^31,32^ can inform the brain whether signals come from a common event and should hence be integrated. This study was designed to investigate to what extent spatial and semantic correspondences influence audiovisual binding in the presence and absence of visual awareness.

Our first experiment demonstrates that the brain combines spatial and semantic correspondences for sound localization. Observers were more accurate to locate the sound when audiovisual signals were collocated relative to when they were presented in opposite hemifields. Critically, semantic congruency amplified audiovisual binding and increased this spatial congruency effect. As indicated by the significant coefficient of the three-way interaction, these semantic influences were more pronounced for bilateral presentation model that induces more complex binding problems (see^38^). Our robust effects of semantic congruency on audiovisual spatial binding contrast with previous research showing that spatial ventriloquism is immune to phonological congruency (^34,35^ but see^38^). We suspect semantic congruency is more effective, because it is computed faster than phonological correspondences that rely on the extraction of fine grained visual features from articulatory movements^64^. In sum, experiment 1 demonstrates that semantic correspondences profoundly influence audiovisual binding for an auditory spatial task.

Experiment 2 next used forward–backward masking to investigate how spatial and semantic congruency influence audiovisual binding depending on observers’ subjective awareness as assessed by perceptual awareness scales. According to leading theories of consciousness, most notably the Global Workspace Theory, one would expect audiovisual binding to be abolished when visual inputs are rendered subjectively invisible. Contrary to this conjecture, we observed a significant—albeit much smaller—spatial ventriloquist effect when the visual inputs were rendered invisible. These results dovetail nicely with previous research showing a robust spatial ventriloquism for flashes that were obliterated from awareness by continuous flash suppression^22^. Collectively, they confirm that visual signals that we are not subjectively aware of can bias where we perceive sounds. The dissociation between the fate of visual inputs in the visual system and their sustained impact on auditory spatial perception may be explained by internal noise along the auditory and visual processing hierarchies^65,66^. This internal noise may stochastically disrupt information transmission selectively along the visual processing hierarchy, even when visual information may impact sound perception via early audiovisual interactions^26,67^. Importantly, however, even though audiovisual binding was to some extent preserved in the absence of awareness, it did no longer depend on semantic congruency. Experiment 2 showed a significant interaction between spatial and semantic congruency only for visible pictures. Bayes factors confirmed the absence of an interaction between spatial and semantic congruency for invisible trials. Collectively, our results thus demonstrate that audiovisual interactions can occur even in the absence of awareness, but these interactions are no longer constrained by higher order semantic correspondences. It is important to emphasize that our results are distinct from previous research showing crossmodal congruency priming^24^ or associative learning^23^ in the absence of awareness. None of those experiments involved integration of sensory information into a unified percept. Instead, their findings can be explained by association and comparison of information from different senses that may be maintained unconsciously in working memory processes as has previously been demonstrated^68–70^. This result, along with studies showing that the McGurk illusion falters under Continuous flash suppression^29,30^, supports the hypothesis that only low (i.e. spatial), but not high level (i.e. semantic) cues can affect multisensory integration in the absence of awareness.

So far, we have shown that semantic congruency did not significantly modulate the influence of an invisible (‘unconscious’) image on observers’ sound localization. Next, we take the opposite perspective and ask whether semantic and/or spatial congruency modulate the influences of a ‘conscious’ sound on observers’ visibility rating or picture identification accuracy. Spatial congruency did not influence observers’ visibility ratings or picture identification accuracy. The absence of spatial constraints on visibility or picture identification accuracy converges with the idea that spatial congruency controls audiovisual binding predominantly in tasks in which spatial information is relevant (e.g. overt or covert spatial orienting—^71–74^) but less so in identification or semantic categorization tasks (e.g.^34^).

As we have already discussed semantic congruency did not modulate the influence of an invisible picture on observers’ sound perception. Semantic congruency non-significantly increased observers’ visibility ratings, a trend that we report in the light of previous findings^3,12,13,18,19^. Thus, a wealth of research has shown that conscious signals in the auditory modality can help semantically congruent signals in the visual modality to elude flash suppression, rivalry suppression^13,15,17^ or the attentional blink^3,12^ suggesting that semantically congruent sounds are more likely to ‘boost’ an otherwise invisible picture into observers’ awareness. In contrast to the weak effect on visibility ratings, observers’ picture identification accuracy was greater when the sound was semantically congruent than incongruent. Contrary to a previously published study^18^ our results thus suggest that increases in picture identification accuracy mainly reflect auditory-induced biases on observers’ responses. When observers cannot identify an invisible picture, they may report the identity of the corresponding sound. As discussed in the introduction, this impact of supraliminal signals in one sensory modality on subliminal processing in another modality can be explained within the Global Workspace Theory, because conscious signals can influence other sensory systems via long-range connectivity throughout the global workspace.

In conclusion, our results demonstrate that spatial and semantic correspondences mould audiovisual interactions flexibly depending on observers’ perceptual goals and awareness. Most notably, conscious and unconscious visual signals can bias where we perceive sounds. Critically, semantic correspondences modulate these crossmodal influences from vision to audition only for pictures that are consciously perceived. This dissociation suggests that semantic correspondences that are computed along the ventral stream are prevented from influencing audiovisual spatial binding along the dorsal stream when visual input is suppressed from visual awareness.

## Supplementary Information

Supplementary Information.

## Data Availability

Data analysed in this study is available on the Open Science Framework repository: https://osf.io/xu2r7/?view_only=f1306ceef4a9494d86f7f7db473ed68e.
