# Supplementary material for: Semantic and spatial congruency mould audiovisual integration depending on perceptual awareness
Source: Sci Rep. 2021 May 25;11:10832. doi: 10.1038/s41598-021-90183-w (PMC8149651; doi:10.1038/s41598-021-90183-w)
Supplement: Supplementary file 1 — Supplementary Information. [file 41598_2021_90183_MOESM1_ESM.docx]

Semantic and spatial congruency mould audiovisual integration depending on perceptual awareness

^1^Patrycja Delong, ^1,2^Uta Noppeney

^1^Centre for Computational Neuroscience and Cognitive Robotics, University of Birmingham, UK

^2^Donders Institute for Brain, Cognition and Behaviour, Radboud University, Nijmegen, Netherlands

## Mean **accuracies in each experimental condition**

| **Condition:** | | | **Mean [%]** | **SEM [%]** |
| --- | --- | --- | --- | --- |
| **Spatial congruency** | **Semantic congruency** | **Presentation mode** |  |  |
| Disparate | Congruent | Unilateral | 20.1 | 19.9 |
| Disparate | Incongruent | Unilateral | 27.3 | 21.7 |
| Collocated | Congruent | Unilateral | 90.2 | 11 |
| Collocated | Incongruent | Unilateral | 88 | 12.6 |
| Disparate | Congruent | Bilateral | 34.3 | 20.1 |
| Disparate | Incongruent | Bilateral | 54.9 | 17.6 |
| Collocated | Congruent | Bilateral | 82.9 | 10.8 |
| Collocated | Incongruent | Bilateral | 71.5 | 11.6 |

**Table 1. Sound localization accuracy in Experiment 1.** Across subjects means and SEM (standard error of the mean).

| **Condition:** | | | **Mean** | **SEM** |
| --- | --- | --- | --- | --- |
| **Spatial congruency** | **Semantic congruency** | **Presentation mode** |  |  |
| Disparate | Congruent | Unilateral | 1.55 | 0.43 |
| Disparate | Incongruent | Unilateral | 1.52 | 0.41 |
| Collocated | Congruent | Unilateral | 1.56 | 0.44 |
| Collocated | Incongruent | Unilateral | 1.51 | 0.41 |
| Disparate | Congruent | Bilateral | 1.56 | 0.41 |
| Disparate | Incongruent | Bilateral | 1.53 | 0.41 |
| Collocated | Congruent | Bilateral | 1.59 | 0.44 |
| Collocated | Incongruent | Bilateral | 1.53 | 0.4 |

**Table 2. Visibility ratings in Experiment 2.** Across subjects means and SEM.

| **Condition:** | | | | **Mean [%]** | **SEM [%]** |
| --- | --- | --- | --- | --- | --- |
| **Spatial congruency** | **Semantic congruency** | **Presentation mode** | **Visibility** |  |  |
| Disparate | Congruent | Unilateral | Visible | 67 | 3.3 |
| Disparate | Incongruent | Unilateral | Visible | 50.2 | 3.7 |
| Collocated | Congruent | Unilateral | Visible | 66.8 | 3.3 |
| Collocated | Incongruent | Unilateral | Visible | 53 | 3.6 |
| Disparate | Congruent | Bilateral | Visible | 65.5 | 3.2 |
| Disparate | Incongruent | Bilateral | Visible | 48.2 | 3.7 |
| Collocated | Congruent | Bilateral | Visible | 62.7 | 3.6 |
| Collocated | Incongruent | Bilateral | Visible | 46.1 | 3.7 |
| Disparate | Congruent | Unilateral | Invisible | 46.2 | 4.9 |
| Disparate | Incongruent | Unilateral | Invisible | 14 | 1.3 |
| Collocated | Congruent | Unilateral | Invisible | 47.2 | 4.8 |
| Collocated | Incongruent | Unilateral | Invisible | 14 | 1.4 |
| Disparate | Congruent | Bilateral | Invisible | 44.5 | 4.5 |
| Disparate | Incongruent | Bilateral | Invisible | 15.2 | 1.3 |
| Collocated | Congruent | Bilateral | Invisible | 44.7 | 4.6 |
| Collocated | Incongruent | Bilateral | Invisible | 15.2 | 1.4 |

**Table 3. Picture identification accuracy in Experiment 2.** Across subjects means and SEM.

| **Condition:** | | | | **Mean [%]** | **SEM [%]** |
| --- | --- | --- | --- | --- | --- |
| **Spatial congruency** | **Semantic congruency** | **Presentation mode** | **Visibility** |  |  |
| Disparate | Congruent | Unilateral | Visible | 53.4 | 3.1 |
| Disparate | Incongruent | Unilateral | Visible | 53 | 2.8 |
| Collocated | Congruent | Unilateral | Visible | 73.5 | 2.2 |
| Collocated | Incongruent | Unilateral | Visible | 66.9 | 2.8 |
| Disparate | Congruent | Bilateral | Visible | 51.7 | 2.9 |
| Disparate | Incongruent | Bilateral | Visible | 51.6 | 3.1 |
| Collocated | Congruent | Bilateral | Visible | 66.9 | 2.9 |
| Collocated | Incongruent | Bilateral | Visible | 67.1 | 2.5 |
| Disparate | Congruent | Unilateral | Invisible | 60.6 | 1.5 |
| Disparate | Incongruent | Unilateral | Invisible | 60.8 | 1.7 |
| Collocated | Congruent | Unilateral | Invisible | 65.7 | 1.8 |
| Collocated | Incongruent | Unilateral | Invisible | 66.4 | 1.7 |
| Disparate | Congruent | Bilateral | Invisible | 59.7 | 1.7 |
| Disparate | Incongruent | Bilateral | Invisible | 59.7 | 1.7 |
| Collocated | Congruent | Bilateral | Invisible | 63 | 1.6 |
| Collocated | Incongruent | Bilateral | Invisible | 63.8 | 1.8 |

**Table 4. Sound localization accuracy in Experiment 2.** Across subjects means and SEM.

**Trial numbers per experimental condition**

| **Condition** | | | Median | Q1 | Q3 | Min | Max |
| --- | --- | --- | --- | --- | --- | --- | --- |
| **Spatial congruency** | **Semantic congruency** | **Presentation mode** |  |  |  |  |  |
| Disparate | Congruent | Unilateral | 80 | 80 | 80 | 69 | 80 |
| Disparate | Incongruent | Unilateral | 80 | 80 | 80 | 70 | 80 |
| Collocated | Congruent | Unilateral | 80 | 80 | 80 | 75 | 80 |
| Collocated | Incongruent | Unilateral | 80 | 80 | 80 | 74 | 80 |
| Disparate | Congruent | Bilateral | 80 | 80 | 80 | 77 | 80 |
| Disparate | Incongruent | Bilateral | 80 | 80 | 80 | 76 | 80 |
| Collocated | Congruent | Bilateral | 80 | 80 | 80 | 78 | 80 |
| Collocated | Incongruent | Bilateral | 80 | 80 | 80 | 79 | 80 |

**Table 5. Number of trials with responses per condition in Experiment 1.** Median, first and third quartile and minimum and maximum values across subjects.

| **Condition** | | | | Median | Q1 | Q3 | Min | Max |
| --- | --- | --- | --- | --- | --- | --- | --- | --- |
| **Spatial congruency** | **Semantic congruency** | **Presentation mode** | **Visibility** |  |  |  |  |  |
| Disparate | Congruent | Unilateral | Visible | 62.5 | 35 | 92 | 0 | 152 |
| Disparate | Incongruent | Unilateral | Visible | 56.5 | 32 | 92.25 | 0 | 141 |
| Collocated | Congruent | Unilateral | Visible | 59 | 34 | 96.25 | 0 | 146 |
| Collocated | Incongruent | Unilateral | Visible | 56 | 33.25 | 89 | 0 | 138 |
| Disparate | Congruent | Bilateral | Visible | 59.5 | 41.5 | 91.5 | 0 | 148 |
| Disparate | Incongruent | Bilateral | Visible | 56.5 | 37.75 | 88.75 | 0 | 150 |
| Collocated | Congruent | Bilateral | Visible | 58 | 41.75 | 93.75 | 1 | 148 |
| Collocated | Incongruent | Bilateral | Visible | 61 | 35.25 | 88.5 | 1 | 147 |
| Disparate | Congruent | Unilateral | Invisible | 96.5 | 68 | 121.5 | 7 | 159 |
| Disparate | Incongruent | Unilateral | Invisible | 103 | 67 | 122.25 | 18 | 159 |
| Collocated | Congruent | Unilateral | Invisible | 101 | 62.75 | 123.25 | 12 | 159 |
| Collocated | Incongruent | Unilateral | Invisible | 104 | 71 | 123 | 21 | 160 |
| Disparate | Congruent | Bilateral | Invisible | 99.5 | 64.5 | 118.5 | 12 | 160 |
| Disparate | Incongruent | Bilateral | Invisible | 103 | 66 | 115.5 | 10 | 160 |
| Collocated | Congruent | Bilateral | Invisible | 100.5 | 60.5 | 118 | 12 | 159 |
| Collocated | Incongruent | Bilateral | Invisible | 96.5 | 67 | 124 | 13 | 158 |

**Table 6. Number of trials with responses per condition in Experiment 2.** Median, first and third quartile and minimum and maximum values across subjects.

## Exploratory analyses of all contrasts for the fitted models

Please note that results are given as log odds ratios. P values were adjusted using Benjamini Hochberg correction.

Experimental conditions in tables below are coded as follows:

Spatial congruency: 1 – collocated, 0 – disparate

Semantic congruency: 1 – congruent, 0 – incongruent

Presentation mode: 1 – bilateral, 0 – unilateral

Visibility: 1 – visible, 0 – invisible

Contrasts (1-0) are given as difference between conditions coded as 1 and 0.

| **Spatial congruency** | **Semantic congruency** | **Presentation mode** | **Coefficient** | **Std. Error** | **z ratio** | **p value** |
| --- | --- | --- | --- | --- | --- | --- |
| **1 - 0** | 0 | 0 | 3.1 | 0.07 | 47.147 | **< 0.001** |
| **1 - 0** | 1 | 0 | 3.75 | 0.07 | 51.878 | **< 0.001** |
| **1 - 0** | 0 | 1 | 0.75 | 0.05 | 14.597 | **< 0.001** |
| **1 - 0** | 1 | 1 | 2.33 | 0.06 | 39.758 | **< 0.001** |
| 0 | **1 - 0** | 0 | -0.42 | 0.06 | -7.259 | **< 0.001** |
| 1 | **1 - 0** | 0 | 0.22 | 0.08 | 2.893 | **0.004** |
| 0 | **1 - 0** | 1 | -0.9 | 0.05 | -17.731 | **< 0.001** |
| 1 | **1 - 0** | 1 | 0.68 | 0.06 | 11.483 | **< 0.001** |
| 0 | 0 | **1 - 0** | 1.25 | 0.05 | 23.759 | **< 0.001** |
| 1 | 0 | **1 - 0** | -1.11 | 0.06 | -17.09 | **< 0.001** |
| 0 | 1 | **1 - 0** | 0.77 | 0.06 | 13.611 | **< 0.001** |
| 1 | 1 | **1 - 0** | -0.65 | 0.07 | -8.958 | **< 0.001** |

**Table 7. All contrasts** for GLMM fitted for **Sound localization accuracy in Experiment 1** (unmasked pictures).

| **Spatial congruency** | **Semantic congruency** | **Presentation mode** | **Coefficient** | **Std. Error** | **z ratio** | **p value** |
| --- | --- | --- | --- | --- | --- | --- |
| **1 - 0** | 0 | 0 | -0.04 | 0.04 | -1.036 | 0.376 |
| **1 - 0** | 1 | 0 | 0.05 | 0.04 | 1.314 | 0.283 |
| **1 - 0** | 0 | 1 | 0.01 | 0.04 | 0.286 | 0.775 |
| **1 - 0** | 1 | 1 | 0.09 | 0.04 | 2.263 | 0.079 |
| 0 | **1 - 0** | 0 | 0.07 | 0.04 | 1.928 | 0.092 |
| 1 | **1 - 0** | 0 | 0.16 | 0.04 | 4.266 | **< 0.001** |
| 0 | **1 - 0** | 1 | 0.08 | 0.04 | 2.221 | 0.079 |
| 1 | **1 - 0** | 1 | 0.16 | 0.04 | 4.197 | **< 0.001** |
| 0 | 0 | **1 - 0** | 0.03 | 0.04 | 0.722 | 0.513 |
| 1 | 0 | **1 - 0** | 0.08 | 0.04 | 2.041 | 0.092 |
| 0 | 1 | **1 - 0** | 0.04 | 0.04 | 1.008 | 0.376 |
| 1 | 1 | **1 - 0** | 0.07 | 0.04 | 1.953 | 0.092 |

**Table 8. All contrasts** for CLMM fitted for **Visibility rating in Experiment 2**.

| **Spatial congruency** | **Semantic congruency** | **Presentation mode** | **Visibility** | **Coefficient** | **Std. Error** | **z ratio** | **p value** |
| --- | --- | --- | --- | --- | --- | --- | --- |
| **1 - 0** | 0 | 0 | 0 | 0.05 | 0.06 | 0.712 | 0.638 |
| **1 - 0** | 1 | 0 | 0 | 0.03 | 0.05 | 0.646 | 0.638 |
| **1 - 0** | 0 | 1 | 0 | -0.01 | 0.06 | -0.208 | 0.891 |
| **1 - 0** | 1 | 1 | 0 | 0.02 | 0.05 | 0.525 | 0.711 |
| **1 - 0** | 0 | 0 | 1 | 0.04 | 0.06 | 0.675 | 0.638 |
| **1 - 0** | 1 | 0 | 1 | 0.04 | 0.06 | 0.649 | 0.638 |
| **1 - 0** | 0 | 1 | 1 | 0.01 | 0.06 | 0.11 | 0.913 |
| **1 - 0** | 1 | 1 | 1 | 0.01 | 0.06 | 0.14 | 0.913 |
| 0 | **1 - 0** | 0 | 0 | 2.07 | 0.06 | 36.615 | **< 0.001** |
| 1 | **1 - 0** | 0 | 0 | 2.06 | 0.06 | 36.601 | **< 0.001** |
| 0 | **1 - 0** | 1 | 0 | 1.57 | 0.05 | 28.672 | **< 0.001** |
| 1 | **1 - 0** | 1 | 0 | 1.61 | 0.05 | 29.269 | **< 0.001** |
| 0 | **1 - 0** | 0 | 1 | 0.55 | 0.06 | 9.49 | **< 0.001** |
| 1 | **1 - 0** | 0 | 1 | 0.55 | 0.06 | 9.397 | **< 0.001** |
| 0 | **1 - 0** | 1 | 1 | 0.71 | 0.06 | 12.343 | **< 0.001** |
| 1 | **1 - 0** | 1 | 1 | 0.71 | 0.06 | 12.444 | **< 0.001** |
| 0 | 0 | **1 - 0** | 0 | 0.24 | 0.06 | 3.789 | **< 0.001** |
| 1 | 0 | **1 - 0** | 0 | 0.18 | 0.06 | 2.887 | **0.006** |
| 0 | 1 | **1 - 0** | 0 | -0.26 | 0.05 | -5.652 | **< 0.001** |
| 1 | 1 | **1 - 0** | 0 | -0.26 | 0.05 | -5.719 | **< 0.001** |
| 0 | 0 | **1 - 0** | 1 | -0.14 | 0.06 | -2.482 | **0.019** |
| 1 | 0 | **1 - 0** | 1 | -0.18 | 0.06 | -3.035 | **0.004** |
| 0 | 1 | **1 - 0** | 1 | 0.02 | 0.06 | 0.265 | 0.886 |
| 1 | 1 | **1 - 0** | 1 | -0.01 | 0.06 | -0.25 | 0.886 |
| 0 | 0 | 0 | **1 - 0** | 1.92 | 0.06 | 30.417 | **< 0.001** |
| 1 | 0 | 0 | **1 - 0** | 1.91 | 0.06 | 30.337 | **< 0.001** |
| 0 | 1 | 0 | **1 - 0** | 0.4 | 0.05 | 7.327 | **< 0.001** |
| 1 | 1 | 0 | **1 - 0** | 0.41 | 0.05 | 7.464 | **< 0.001** |
| 0 | 0 | 1 | **1 - 0** | 1.53 | 0.06 | 25.176 | **< 0.001** |
| 1 | 0 | 1 | **1 - 0** | 1.55 | 0.06 | 25.546 | **< 0.001** |
| 0 | 1 | 1 | **1 - 0** | 0.67 | 0.05 | 12.441 | **< 0.001** |
| 1 | 1 | 1 | **1 - 0** | 0.66 | 0.05 | 12.159 | **< 0.001** |

**Table 9. All contrasts** for GLMM fitted for **Picture identification accuracy in Experiment 2**.

| **Spatial congruency** | **Semantic congruency** | **Presentation mode** | **Visibility** | **Coefficient** | **Std. Error** | **z ratio** | **p value** |
| --- | --- | --- | --- | --- | --- | --- | --- |
| **1 - 0** | 0 | 0 | 0 | 0.21 | 0.05 | 4.685 | **< 0.001** |
| **1 - 0** | 1 | 0 | 0 | 0.16 | 0.05 | 3.542 | **0.001** |
| **1 - 0** | 0 | 1 | 0 | 0.18 | 0.05 | 3.983 | **< 0.001** |
| **1 - 0** | 1 | 1 | 0 | 0.08 | 0.05 | 1.702 | 0.142 |
| **1 - 0** | 0 | 0 | 1 | 0.67 | 0.06 | 11.461 | **< 0.001** |
| **1 - 0** | 1 | 0 | 1 | 0.85 | 0.06 | 14.632 | **< 0.001** |
| **1 - 0** | 0 | 1 | 1 | 0.66 | 0.06 | 11.498 | **< 0.001** |
| **1 - 0** | 1 | 1 | 1 | 0.74 | 0.06 | 12.995 | **< 0.001** |
| 0 | **1 - 0** | 0 | 0 | 0.02 | 0.05 | 0.501 | 0.657 |
| 1 | **1 - 0** | 0 | 0 | -0.03 | 0.05 | -0.603 | 0.603 |
| 0 | **1 - 0** | 1 | 0 | 0.04 | 0.05 | 0.92 | 0.409 |
| 1 | **1 - 0** | 1 | 0 | -0.06 | 0.05 | -1.339 | 0.262 |
| 0 | **1 - 0** | 0 | 1 | -0.02 | 0.06 | -0.362 | 0.718 |
| 1 | **1 - 0** | 0 | 1 | 0.17 | 0.06 | 2.77 | **0.011** |
| 0 | **1 - 0** | 1 | 1 | -0.02 | 0.06 | -0.361 | 0.718 |
| 1 | **1 - 0** | 1 | 1 | 0.06 | 0.06 | 1.021 | 0.364 |
| 0 | 0 | **1 - 0** | 0 | -0.07 | 0.05 | -1.524 | 0.194 |
| 1 | 0 | **1 - 0** | 0 | -0.1 | 0.05 | -2.202 | **0.049** |
| 0 | 1 | **1 - 0** | 0 | -0.05 | 0.05 | -1.098 | 0.34 |
| 1 | 1 | **1 - 0** | 0 | -0.14 | 0.05 | -2.911 | **0.008** |
| 0 | 0 | **1 - 0** | 1 | -0.06 | 0.06 | -1.088 | 0.34 |
| 1 | 0 | **1 - 0** | 1 | -0.07 | 0.06 | -1.171 | 0.336 |
| 0 | 1 | **1 - 0** | 1 | -0.06 | 0.06 | -1.094 | 0.34 |
| 1 | 1 | **1 - 0** | 1 | -0.18 | 0.06 | -2.981 | **0.007** |
| 0 | 0 | 0 | **1 - 0** | -0.36 | 0.05 | -6.867 | **< 0.001** |
| 1 | 0 | 0 | **1 - 0** | 0.1 | 0.05 | 1.734 | 0.14 |
| 0 | 1 | 0 | **1 - 0** | -0.4 | 0.05 | -7.651 | **< 0.001** |
| 1 | 1 | 0 | **1 - 0** | 0.29 | 0.06 | 5.225 | **< 0.001** |
| 0 | 0 | 1 | **1 - 0** | -0.35 | 0.05 | -6.736 | **< 0.001** |
| 1 | 0 | 1 | **1 - 0** | 0.13 | 0.05 | 2.357 | **0.035** |
| 0 | 1 | 1 | **1 - 0** | -0.41 | 0.05 | -7.921 | **< 0.001** |
| 1 | 1 | 1 | **1 - 0** | 0.25 | 0.05 | 4.602 | **< 0.001** |

**Table 10. All contrasts** for GLMM fitted for **Sound localisation accuracy in Experiment 2**.
